# Annual Acknowledgement of Reviewers

**DOI:** 10.1186/s12918-016-0272-5

**Published:** 2016-03-15

**Authors:** Timothy R. Sands

**Affiliations:** BioMed Central, Floor 6, 236 Gray’s Inn Road, London, WC1X 8HB UK

## Abstract

The editors of BMC Systems Biology would like to thank all our reviewers who have contributed to the journal in Volume 9 (2015).

**Enzo Acerbi**

Singapore

**Peter Ahnert**

Germany

**Tobias Ahsendorf**

Germany

**Uri David Akavia**

Canada

**Réka Albert**

USA

**Eivind Almaas**

Norway

**David Arnosti**

USA

**Michael Assaf**

Israel

**Fazoil Ataullakhanov**

USA

**Rejia Autio**

Finland

**Kambiz Baghalian**

UK

**Francesca Ballarini**

USA

**Julio Banga**

Spain

**Debmalya Barh**

India

**Dorjsuren Battogtokh**

USA

**Jan Baumbach**

Denmark

**David Baumler**

USA

**Eddy Bautista**

USA

**Robert Bender**

USA

**Kobi Benenson**

USA

**Alfredo Benso**

Italy

**Jonathan Berrout**

UK

**Francois Berthiaume**

USA

**Fortunato Bianconi**

Italy

**Franco Blanchini**

Italy

**Michael Blinov**

USA

**Xiaochen Bo**

China

**Jorge Boczkowski**

France

**Gib Bogle**

New Zealand

**Aarash Bordbar**

USA

**Dragan Bosnacki**

Netherlands

**Jérémie Bourdon**

France

**Filipe Branco Dos Santos**

Netherlands

**Yury Bukhman**

USA

**Jake Bundy**

UK

**Hauke Busch**

Germany

**Rongman Cai**

USA

**Michael Calderwood**

USA

**Laurence Calzone**

France

**Pablo Carbonell**

UK

**Gunnar Cedersund**

Sweden

**Sriram Chandrasekaran**

USA

**Arvind Chavali**

USA

**Chien-Hsiun Chen**

Taiwan

**Xing Chen**

China

**Chun Chen**

USA

**Guocai Chen**

USA

**Leonid Chindelevitch**

USA

**Eugenio Cinquemani**

France

**Darren Creek**

Australia

**Qinghua Cui**

China

**Tobias Czauderna**

Australia

**Joseph Dada**

Nigeria

**Lorenza D'Allesandro**

Germany

**Thomas Dandekar**

Germany

**Andrea De Martino**

Italy

**Barry Demchak**

USA

**Sunil Deshpande**

USA

**Luigi Di Costanzo**

USA

**Allan Dickerman**

USA

**Elena Dimitrova**

USA

**Josef Donnerer**

Austria

**Andreas Dräger**

Germany

**Bin Du**

USA

**Ilija Dukovsky**

USA

**Warwick Dunn**

UK

**Saliha Durmuş Tekir**

Turkey

**Johann Eicher**

South Africa

**Frank Emmert-Streib**

Finland

**Sertac Eroglu**

Turkey

**Fazle Faisal**

USA

**Adrien Fauré**

Japan

**Anton Feenstra**

Netherlands

**David Fell**

UK

**Kathrin Fenner**

Switzerland

**Zelia Ferreira**

USA

**Dirk Fey**

Ireland

**Marc Thilo Figge**

Germany

**Gianfranco Fiore**

UK

**Ben Fitzpatrick**

USA

**Ronan Fleming**

Luxembourg

**Stephen Fong**

USA

**Laura Furlong**

Spain

**Rui Gan**

Canada

**Erol Gelenbe**

UK

**Patrick Gillevet**

USA

**Enrico Glaab**

Luxembourg

**Ingmar Glauche**

Germany

**Haijun Gong**

USA

**Assaf Gottlieb**

USA

**Ramon Grima**

UK

**Rudiyanto Gunawan**

Switzerland

**Shailendra Gupta**

Germany

**Juergen Hahn**

USA

**William Harcombe**

USA

**Heather Harrington**

UK

**Jan Hasenauer**

Germany

**Benjamin Heavner**

USA

**Tomas Helikar**

USA

**Volkhard Helms**

Germany

**Markus Herrgard**

Denmark

**Steven Hill**

UK

**William Holmes**

USA

**Antti Honkela**

Finland

**Hsuan-Cheng Huang**

Taiwan

**Yue Huang**

USA

**Dagmar Iber**

Switzerland

**Kimiyoshi Ichida**

Japan

**Mark Isalan**

UK

**Zerrin Isik**

Turkey

**Boris Ivanov**

Russia

**Bayu Jayawardhana**

Netherlands

**Jong Cheol Jeong**

USA

**Yi Jiang**

USA

**Anagha Joshi**

UK

**Stefan Kallenberger**

Germany

**Sandip Kar**

India

**Petros Karakousis**

USA

**Jonathan Karr**

USA

**Michael Katze**

USA

**Evgeniy Khain**

USA

**Andrzej Kierzek**

UK

**Yon Hui Kim**

South Korea

**Tamara Kinzer-Ursem**

USA

**Akihiko Kondo**

Japan

**Rainer Konig**

Germany

**Matthias König**

Germany

**Andreas Kremling**

Germany

**Zheng Kuang**

USA

**Robert Küffner**

Germany

**Martin Kuiper**

Norway

**Don Kulasiri**

New Zealand

**Lekha Kumar**

India

**Yasutaka Kurata**

Japan

**Istvan Ladunga**

USA

**Camille Laibe**

UK

**Sandro Lambeck**

Germany

**Kerry Landman**

Australia

**Mario Latendresse**

USA

**Alan Yueh-Luen Lee**

Taiwan

**Dong-Yup Lee**

Singapore

**Jennifer Levering**

USA

**Nathan Lewis**

USA

**Jingjing Li**

USA

**Pu Li**

Germany

**Fangting Li**

China

**Haomin Li**

China

**Jie Liang**

USA

**Gabriele Lillacci**

Switzerland

**Hao Lin**

China

**Jennifer Linderman**

USA

**Haiyan Liu**

China

**Rui Liu**

China

**Fernando Lopez-Caamal**

Mexico

**Mingyang Lu**

USA

**Timo Lubitz**

Germany

**Jamie Luo**

UK

**Feng Luo**

USA

**Stuart Macdonald**

USA

**Daniel Machado**

Portugal

**Adam Maclean**

UK

**William MacNee**

UK

**Rosalinda Madonna**

Italy

**Tim Maiwald**

Germany

**Kristiina Mäkinen**

Finland

**Ani Manichaikul**

USA

**Costas Maranas**

USA

**Luca Marchetti**

Italy

**Florian Markowetz**

Germany

**Carsten Marr**

Germany

**Tommaso Mazza**

Italy

**Jason McDermott**

USA

**Joerg Menche**

Hungary

**Pedro Mendes**

UK

**Pablo Meyer**

USA

**Pejman Mohammadi**

USA

**Jonathan Monk**

USA

**John Morgan**

USA

**John Morris**

USA

**Richard Morris**

UK

**Mark Muldoon**

UK

**Kenta Nakai**

Japan

**Ali Navid**

USA

**Hadi Nazem-Bokaee**

USA

**David Nickerson**

New Zealand

**Stephan Noack**

Germany

**Graham Noctor**

France

**Juan Nogales**

Spain

**Daniel Noguera**

USA

**Joshua Obar**

USA

**Matthew Oberhardt**

Israel

**Masahiro Ogawa**

Japan

**Brett Olivier**

Netherlands

**Catharina Olsen**

Belgium

**Zoltan Oltvai**

USA

**Nicolas Orsi**

UK

**Irene Otero Muras**

Spain

**Alida Palmisano**

USA

**Di Pan**

USA

**Jason Papin**

USA

**Balazs Papp**

Hungary

**Daniele Pepe**

Belgium

**Silvina Ponce Dawson**

Argentina

**Julia Ponomarenko**

Spain

**Gibin Powathil**

UK

**Nicole Erika Radde**

Germany

**Ovidiu Radulescu**

France

**Karthik Raman**

India

**Nicholas Rattray**

UK

**Jennifer Reed**

USA

**Elisabeth Remy**

France

**Guillaume Rey**

UK

**Zweigerdt Robert**

Germany

**Miriam Rodriguez**

Spain

**Marianne Rooman**

Belgium

**Eytan Ruppin**

Israel

**Maria Pia Saccomani**

Italy

**Julio Saez-Rodriguez**

UK

**Supreet Saini**

India

**Leonor Saiz**

USA

**Areejit Samal**

Germany

**Maria Samsonova**

Russia

**Julia Sanwald**

Germany

**Sibaji Sarkar**

USA

**Herbert Sauro**

USA

**Marcel Schilling**

Germany

**Martin Schuster**

USA

**Mary Sehl**

USA

**Ryan Senger**

USA

**Velia Siciliano**

USA

**Evangelos Simeonidis**

USA

**Kieran Smallbone**

UK

**Lucian Smith**

USA

**Daniel Sobral**

Portugal

**Michael Sokolov**

Switzerland

**Sylvain Soliman**

France

**Björn Sommer**

Germany

**Lei Song**

USA

**Nikolaus Sonnenschein**

Denmark

**Vonwun Soo**

Taiwan

**Natalie Stanford**

UK

**Melanie Stefan**

UK

**Dov Stekel**

UK

**Ralf Steuer**

Germany

**Mark Styczynski**

USA

**Gang Su**

USA

**Jingchun Sun**

USA

**Neil Swainston**

UK

**Bas Teusink**

Netherlands

**Chabane Tibiche**

Canada

**Metin Turkay**

Turkey

**Jannis Uhlendorf**

Germany

**Adelinde Uhrmacher**

Germany

**Hyun Uk Kim**

South Korea

**Rajanikanth Vadigepalli**

USA

**David Van Dijk**

Israel

**Joep Vanlier**

Germany

**Alan Veliz-Cuba**

USA

**Peter Verheijen**

Netherlands

**Ganesh Viswanathan**

India

**Steffen Waldherr**

Germany

**Dagmar Waltemath**

Germany

**Tao Wang**

USA

**Tyisha Williams**

USA

**John Williams**

USA

**Bridget Wilson**

USA

**Limsoon Wong**

Singapore

**Ming Wu**

USA

**Wei-Sheng Wu**

Taiwan

**Wenting Wu**

USA

**Lingyun Wu**

China

**Michelle Wynn**

USA

**Jianhua Xing**

USA

**Xuerui Yang**

China

**Laurence Yang**

USA

**Yanjun Yang**

China

**Guang Yao**

USA

**Phillip Yen**

USA

**Sung Ho Yoon**

South Korea

**William Young**

USA

**Jiyang Yu**

USA

**Mattia Zampieri**

Switzerland

**Christoph Zechner**

Switzerland

**Tao Zeng**

China

**Tongli Zhang**

UK

**Shu-Dong Zhang**

UK

**Tongli Zhang**

USA

**Yuhua Zhao**

China

**Hufeng Zhou**

Singapore

**Hong Zhu**

USA

**Xiaofeng Zhu**

USA

**Daniel Zielinski**

USA

**Andrei Zinovyev**

France

